# Prostitution of High Health

**Published:** 1882-07

**Authors:** 


### PROSTITUTION OF HIGH HEALTH.

NEVER having read Shakspeare, or Milton, or Byron, or the productions of the "Great Unknown," the presumption is that we have missed a great deal or nothing. Schiller is the high-priest of some, and we have heard Emerson quote Goethe, as if he were a god in his estimation. The life of the "Patriarch of German Literature," was one of great contradictions. Before he was eight years old, he was exercising in German, French, Italian, Latin, and Greek, yet did not die of brain-fever or dropsy in the head. He drank wine, was prodigal of money, and was odd in his manners. His "social faults looked society contemptuously in the face," but as a swordsman, a rider, and a skater, beyond most of his time, he lived to the age of eighty-two years. He wrote one of the most doleful books ever published, known as the *Sorrows of Werther*, yet himself took life without sadness, and enjoyed it to the full. His writings gave out a light of their kind, in the glare of which multitudes delight still to live; yet at the age of sixty he doubted the existence of a divine providence, because he could not see through His dispensations, who in wisdom ruleth over all, and at the age of eighty-two, he still could not see, and died exclaiming: "More light!" He died in darkness when the world was in a blaze of sunshine! And why? because he was without a Bible, without a religious faith, and without a God, if we take his favorite expression as the embodiment of his creed,

"The end of life is life itself,"

which, if it means any thing, is,

*Let us eat and drink, for to-morrow we die.*

Goethe had high health, a vigorous constitution, a great position, and a world of friends. But with the baleful sentiment that this life was every thing, God was not in all his thoughts, and he lived only in himself.

This narration is given as a warning to the few who never have an ache or a pain, that they may not grow up and live thankless and thoughtless of the great talent of perfect health, but rather consecrate it, in humility, to good-doing, so that when the mortal eye closes in the night of the grave, it may open upon the ineffable splendors of an immortal existence.

